# Prevalence and diagnosis experience of osteoporosis in postmenopausal women over 50: Focusing on socioeconomic factors

**DOI:** 10.1371/journal.pone.0248020

**Published:** 2021-03-02

**Authors:** Min Hyeok Choi, Ji Hee Yang, Jae Seung Seo, Yoon-ji Kim, Suk-Woong Kang

**Affiliations:** 1 Department of Preventive and Occupational Medicine, Pusan National University Yangsan Hospital, Yangsan, Republic of Korea; 2 Department of Medicine, Pusan National University, Busan, Republic of Korea; 3 Department of Orthopedics, Pusan National University Yangsan Hospital, Yangsan, Republic of Korea; Universidad de Extremadura Facultad de Enfermeria y Terapia Ocupacional, SPAIN

## Abstract

Osteoporosis is the most common disease of the musculoskeletal system in old age. Therefore, research on osteoporosis risk factors is actively being conducted. However, whether socioeconomic inequality is associated with the prevalence and diagnosis experience of osteoporosis remains largely unexplored. This study aims to investigate whether socioeconomic inequality can be a risk factor for osteoporosis in postmenopausal women. Cross-sectional data of 1,477 postmenopausal women aged over 50 obtained from the Korea National Health and Nutrition Examination Survey V-2 were analyzed. Univariate analyses were performed to calculate the prevalence of osteoporosis and the rate of osteoporosis diagnosis experience according to the risk factor categories. Multivariate logistic regression analysis was performed to identify the independent variables’ associations with osteoporosis prevalence and diagnosis experience. The prevalence of osteoporosis was 34.8%, while the diagnosis experience rate was 22.1%. The higher the age, the higher the probability of osteoporosis presence and diagnosis experience. The lowest household income level was associated with a 1.63 times higher risk of osteoporosis. On the contrary, this factor was not significant for diagnosis experience. These results were similar for the 50–59 and 60–69 age groups. Among postmenopausal women, those who are older and have low socioeconomic levels are at a high risk of developing osteoporosis. Moreover, the lower the socioeconomic level, the lower the awareness of osteoporosis. Therefore, there is a need to develop more proactive preventive measures in postmenopausal women with low socioeconomic levels.

## Introduction

Osteoporosis is a common musculoskeletal disease associated with reduced bone strength and disruption of bone architecture [1]. In 1994 and 2008, the World Health Organization published diagnostic criteria for osteoporosis in postmenopausal women based on the T-score for bone mineral density (BMD). According to these criteria, osteoporosis is defined as a BMD value 2.5 standard deviations or more below the young female adult mean (T-score ≤ -2.5 standard deviations) [2, 3].

Although usually asymptomatic, osteoporosis is the most common cause of fractures in older adults [2, 4]. The consequences of osteoporotic fractures include severe morbidity, disability, poor quality of life, and mortality [5]. Furthermore, as a result of the surgical treatment and prolongation of hospitalization associated with osteoporotic fracture-related complications, the economic burden of osteoporosis is on the rise and is increasingly being recognized as a serious public health problem [6, 7].

Owing to the global interest in osteoporosis risk, research on risk factors is underway [8, 9]. It has been reported that osteoporosis can result from a variety of causes, such as family history, chronic diseases, and environmental factors. Some of these risk factors for osteoporosis can be controlled by the individual, while others cannot [10–12].

It has been suggested that socioeconomic disparities can lead to chronic diseases. In various studies, diabetes, stroke, heart disease, and cancer showed differences in incidence rates according to the income gap [13–15]. In the context of rising socioeconomic inequality, which is difficult to address at the individual level, social responsibility is warranted.

There are several studies investigating the influence of socioeconomic level on the prevalence of osteoporosis [16–18]. However, there is a dearth of reports comparing the effects of different socioeconomic levels on the prevalence or diagnosis of osteoporosis. Therefore, the purpose of this study is to:

1. Calculate and compare the prevalence and diagnosis experience rates of osteoporosis in postmenopausal women aged over 50.
2. Identify and compare the associations of socioeconomic factors, focusing on household income level, with the prevalence and diagnosis experience of osteoporosis.
3. Examine the implications of an osteoporosis control policy based on the evidence obtained.

## Materials and methods

### Data and study population

Data were obtained from the Korea National Health and Nutrition Examination Survey (KNHANES) V-2. Since 1998, the Korea Disease Control and Prevention Agency has annually conducted the KNHANES, which produces an index of health behavior, nutrition, and prevalence of chronic diseases in the country. The findings are being used as the basis of a national health plan [19]. Every year, the survey is performed in 192 districts with 3,800 households composed of individuals aged one and above. Standardized physical examinations for various diseases and interview surveys on health behaviors, nutrition, and socioeconomic status are conducted according to the participants’ life cycles. The KNHANES dataset is one of the largest in the country, facilitating research on osteoporosis based on a nationally representative sample. In the present study, among the participants of the KNHANES V-2, 1,477 postmenopausal women aged over 50 who had undergone BMD testing were analyzed.

### Variables

The outcome variables in this study were the presence and diagnosis of osteoporosis. The presence or absence of osteoporosis was determined based on T-scores obtained from the BMD measurement of the total femur, femoral neck, and spine with dual-energy X-ray absorptiometry (DEXA; Hologic Discovery, Hologic, Marlborough, MA, USA). The precision value of the equipment, in terms of the coefficient of variation (CV), was 1.9% for the spine, 1.8% for the total femur and 2.5% for the femoral neck. This value was obtained by scanning 30 randomly selected subjects who underwent two scans on the same day while getting off and back onto the examination table between their examinations. The T-score is most commonly used to identify osteoporosis and determine fracture risk. In this study, cases with a T-score of ≤ -2.5 were considered to have osteoporosis [2, 3]. The presence of diagnosis experience was based on a “Yes” response to the question “Have you ever been diagnosed with osteoporosis by a medical doctor?” in the interview.

To identify and compare factors associated with the presence of osteoporosis and diagnosis experience, age (50–59 years, 60–69 years, ≥ 70 years), educational level (middle school or below, high school graduate, university graduate or above), equivalised household income (1st [highest level]–5th [lowest level] quartile), high-risk drinking (drinking alcohol ≥ 2 times a week, five drinks at a time), physical activity (vigorous physical activity for ≥ 20 minutes three days a week, or moderate physical activity ≥ 30 minutes, five days in the last week), hypertension (systolic blood pressure ≥ 140mmHg, diastolic blood pressure ≥ 90mmHg, or hypertension medication), and diabetes (fasting blood sugar ≥ 126mg/dL or diabetes medication), which have either previously been reported as risk factors for osteoporosis or were to be explored in this study, were included as independent variables [1–5].

### Statistical analysis

The prevalence of osteoporosis and rate of diagnosis experience for each independent variable category were calculated using univariate analysis, and an χ^2^ test was performed to determine statistical significance. Multivariate logistic regression analyses were performed to identify the associations of individual factors after adjusting for other factors. In addition, multivariate logistic regression analyses by age group were performed to determine the association of equivalised household income level with osteoporosis prevalence and diagnosis experience. Two-tailed *p*-values < 0.05 were considered statistically significant. All statistical analyses were performed using STATA MP 15.1 (Stata Corporation, College Station, TX, USA).

### Ethical considerations

The present study was approved by the Institutional Review Board of Pusan National University Hospital (IRB No. H-1901-019-075).

## Results

### General characteristics of the study population

Table 1 shows the baseline characteristics of the subjects. Women aged 50–59 formed the majority of the sample. Further, most subjects had an educational level of middle school or below, and the 5th quartile of household income was the most common. In addition, 1.2% of the sample was categorized into the high-risk drinking group, 20.1% engaged in physical activity, and the prevalence rates of hypertension and diabetes were 51.3% and 13.5%, respectively.

**Table 1:** General characteristics of the study population.

		N	%
**Total**		1,477	100.0
**Age group**	50–59	552	37.4
	60–69	505	34.2
	≥ 70	420	28.4
**Educational level**	Middle school or below	1,184	80.2
	High school	229	15.5
	University or above	64	4.3
**Household income level**^a^	1 (highest)	218	14.8
	2	154	10.4
	3	264	17.9
	4	256	17.3
	5 (lowest)	585	39.6
**High-risk drinking**^b^	No	1,459	98.8
	Yes	18	1.2
**Physical activity**^c^	No	1,180	79.9
	Yes	297	20.1
**Obesity**^d^	No	1,407	95.3
	Yes	70	4.7
**Hypertension**^e^	No	720	48.7
	Yes	757	51.3
**Diabetes**^f^	No	1,278	86.5
	Yes	199	

^a^Categorized using equivalised income

^b^drinking alcohol ≥ 2 times a week, five drinks at a time

^c^engaging in vigorous physical activity for ≥ 20 minutes thrice a week, or moderate physical activity ≥ 30 minutes, five days in the last week

^d^body mass index ≥ 30 kg/m^2^

^e^systolic blood pressure ≥ 140mmHg, diastolic blood pressure ≥ 90mmHg or hypertension medication

^f^fasting blood sugar ≥ 126mg/dL or diabetes medication.

### Osteoporosis prevalence and diagnosis experience

Table 2 shows the prevalence of osteoporosis and the diagnosis experience rate by variable categories. The overall prevalence of osteoporosis and the diagnosis experience rate was 34.8% and 22.1%, respectively. Among the subjects, 29.6% of those with T-scores ≤ -2.5 (n = 514) had diagnosis experience. By age group, both prevalence and diagnosis experience rates were highest among those aged over 70. By household income level, both prevalence and diagnosis experience rates were highest at level 5; this result was statistically significant (*p* < 0.05). Further, at the same household income level, the prevalence rate was higher than the diagnosis experience rate. In particular, at the lowest household income level, the difference between the diagnosis experience and prevalence rates was the largest (18.1%).

**Table 2:** Osteoporosis prevalence and diagnosis experience rates by individual factors.

		Prevalence			Diagnosis experience		
		n	%	χ^2^ test	n	%	χ^2^ test
							*p*-value
				*p*-value			
**Total**		514	34.8	-	326	22.1	-
**Age group**	50–59	82	14.9	< 0.001	61	11.1	< 0.001
	60–69	165	32.7		126	25.0	
	≥ 70	267	63.6		139	33.1	
**Educational level**	Middle school or below	473	39.9	< 0.001	275	23.2	0.095
	High school	32	14.0		39	17.0	
	University or above	9	14.1		12	18.8	
**Household income level**	1 (highest)	44	20.2	< 0.001	34	15.6	0.001
	2	37	24.0		24	15.6	
	3	76	28.8		54	20.5	
	4	92	35.9		55	21.5	
	5 (lowest)	265	45.3		159	27.2	
**High-risk drinking**	No	511	35.0	0.104	321	22.0	0.557
	Yes	3	16.7		5	27.8	
**Physical activity**	No	427	36.2	0.026	253	21.4	0.244
	Yes	87	29.3		73	24.6	
**Obesity**	No	501	35.6	0.003	315	22.4	0.189
	Yes	13	18.6		11	15.7	
**Hypertension**	No	224	31.1	0.004	151	21.0	0.320
	Yes	290	38.3		175	23.1	
**Diabetes**	No	454	35.5	0.139	273	21.4	0.095
	Yes	60	30.2		53	26.6	

### Association of individual factors with osteoporosis prevalence and diagnosis experience rate

Table 3 shows the results of analyzing the associations of individual factors while controlling for other variables. When prevalence was used as an outcome variable, the significant variables were age group, household income level, obesity, and diabetes (*p* < 0.05). Osteoporosis risk increased with age; the probability of developing osteoporosis was 8.47 times higher in the group aged 70 or older (95% confidence interval [CI] 6.01–11.94, *p* < 0.05) compared to the 50–59 group. The adjusted odds ratios (ORs) of the groups with the lowest household income were 1.66 (95% CI 1.04–2.64, *p* = 0.032) for level 4 and 1.63 (95% CI 1.07–2.48, *p* = 0.022) for level 5; these were statistically significant. The ORs of the groups with obesity and diabetes were 0.33 (95% CI 0.17–0.63, *p* = 0.001) and 0.53 (95% CI 0.37–0.76, *p* = 0.001), respectively. However, there were no factors relevant to diagnosis experience rate except age group.

**Table 3:** Association of individual factors with osteoporosis prevalence and diagnosis experience using multivariate logistic regression analysis.

		Prevalence			Diagnosis experience		
		Adj, OR	(95% CI)	*p-*value	Adj, OR	(95% CI)	*p-*value
**Age group**	50–59	Reference			Reference		
	60–69	2.40	(1.74–3.31)	< 0.001	2.72	(1.90–3.90)	< 0.001
	≥ 70	8.47	(6.01–11.94)	< 0.001	4.20	(2.87–6.13)	< 0.001
**Educational level**	Middle school or below	Reference			Reference		
	High school	0.85	(0.37–1.97)	0.704	0.77	(0.37–1.62)	0.497
	University or above	2.11	(0.98–4.52)	0.056	0.71	(0.35–1.41)	0.323
**Household income level**	1 (highest)	Reference			Reference		
	2	1.24	(0.72–2.14)	0.434	1.08	(0.60–1.94)	0.804
	3	1.47	(0.92–2.35)	0.105	1.36	(0.83–2.21)	0.220
	4	1.66	(1.04–2.64)	0.032	1.30	(0.79–2.14)	0.296
	5 (lowest)	1.63	(1.07–2.48)	0.022	1.40	(0.90–2.17)	0.139
**High-risk drinking**	No	Reference			Reference		
	Yes	0.64	(0.17–2.46)	0.516	2.36	(0.78–7.10)	0.128
**Physical activity**	No	Reference			Reference		
	Yes	0.80	(0.59–1.09)	0.166	1.34	(0.98–1.83)	0.065
**Obesity**	No	Reference			Reference		
	Yes	0.33	(0.17–0.63)	0.001	0.59	(0.30–1.15)	0.120
**Hypertension**	No	Reference			Reference		
	Yes	0.93	(0.72–1.20)	0.568	0.86	(0.66–1.13)	0.278
**Diabetes**	No	Reference			Reference		
	Yes	0.53	(0.37–0.76)	0.001	1.17	(0.82–1.67)	0.388

Adj OR: adjusted odds ratio; CI: confidence interval.

### Associations of household income level by age group

Table 4 shows the results of multivariate logistic regression analysis performed to determine the association of household income level, according to age group, with osteoporosis prevalence and diagnosis experience. Among subjects with low household income, the OR was significantly higher among those aged 50–69 (level 4 OR = 2.27) and 60–69 (level 5 OR = 2.06) compared to those with the highest household income level. On the contrary, the role of household income level was not confirmed in subjects aged over 70. Unlike the case with osteoporosis prevalence, household income level was not significantly associated with diagnosis experience.

**Table 4:** Association of household income level with osteoporosis prevalence and diagnosis experience by age group using multivariate logistic regression analysis.

Household income level		50–59 years		60–69 years		≥ 70 years	
		Adj OR (95% CI)	*p-*value	Adj OR (95% CI)	*p-*value	Adj OR (95% CI)	*p-*value
**Prevalence**							
	Level 1 (highest)	Reference		Reference		Reference	
	Level 2	1.11 (0.46–2.67)	0.823	0.78 (0.26–2.40)	0.668	2.91 (0.90–9.41)	0.074
	Level 3	1.92 (0.89–4.13)	0.096	1.26 (0.55–2.85)	0.585	2.85 (0.59–1.32)	0.567
	Level 4	2.27* (1.03–5.03)	0.042	1.27 (0.56–2.86)	0.569	2.86 (0.57–1.90)	0.164
	Level 5 (lowest)	1.58 (0.69–3.63)	0.283	2.06* (1.00–4.22)	0.049	4.22 (0.05–1.08)	0.831
**Diagnosis experience**							
	Level 1 (highest)	Reference		Reference		Reference	
	Level 2	1.56 (0.60–4.06)	0.358	1.15 (0.39–3.39)	0.800	3.39 (0.80–0.54)	0.294
	Level 3	2.21 (0.95–5.16)	0.067	0.94 (0.40–2.21)	0.896	2.21 (0.90–1.09)	0.850
	Level 4	1.67 (0.65–4.26)	0.287	1.49 (0.65–3.41)	0.344	3.41 (0.34–0.77)	0.574
	Level 5 (lowest)	1.91 (0.74–4.89)	0.180	1.38 (0.66–2.88)	0.392	2.88 (0.39–0.98)	0.958

Adjusted for educational level, high-risk drinking, physical activity, obesity, hypertension, and diabetes.

Adj OR: adjusted odds ratio; CI: confidence interval.

## Discussion

In this study of postmenopausal women aged over 50, 34.8% had osteoporosis and 22.1% had been diagnosed with osteoporosis. Significant correlates of osteoporosis prevalence were age, household income level, obesity, and diabetes. Furthermore, the probability of the presence of osteoporosis was about 1.6 times higher in the low household income group (levels 4 and 5) compared to the highest household income group. However, there was no difference in diagnosis experience, implying that overall, there were fewer opportunities for diagnosis in those with low socioeconomic levels.

In many countries, studies on the prevalence of osteoporosis are actively being conducted. The prevalence of osteoporosis in China has increased over the past 12 years, affecting more than one-third of people aged 50 and above [20]. In the European Union, 21% of women aged 50–84 years had osteoporosis in 2010 [6]. In a Turkish study, the prevalence of osteoporosis at the femoral neck was 7.5% and 33.3% in men and women aged 50 and above, respectively [21]. In an American study conducted in 2010, 10.3% of those aged 50 and above (numbering 10.2 million) had osteoporosis at the femoral neck or lumbar spine and 43.9% (43.4 million) had low bone mass at either skeletal site [22]. The results of the present study are similar, with a 34.8% prevalence of osteoporosis in women aged over 50.

Multidisciplinary analyses have reported various risk factors for osteoporosis [1–5]. Old age is the greatest risk factor; with a 2.4 times higher prevalence rate in women over 60 and an 8.5 times higher prevalence rate in women over 70, this was observed in the present study as well. In our study, cases with diabetes and obesity, however, had a lower risk of osteoporosis than those without these conditions. The effects of obesity and diabetes observed in this study were consistent with the results of some prior studies, while they contradicted those of others [2, 8, 23–25]. In the past, some studies have showed that higher body weight can slow down menopausal bone loss [26]. Furthermore, studies have reported lower hip fracture rates among older adults with obesity; this is because of the cushioning effect of body fat on bony eminences [27, 28]. However, recent studies have not considered obesity a prophylactic factor for osteoporosis because of its potential long-term adverse effects, such as on bone quality [29]. In addition, conflicting results have been reported on the relationship between osteoporosis and diabetes, especially type 2 diabetes mellitus [30, 31]. Although the prevalence of type 2 diabetes in South Korea has increased [32], our analysis was limited by the lack of information about diabetes type. Therefore, a long-term follow-up study is needed to verify the relationship between diabetes and obesity.

In general, there are limited studies exploring osteoporosis in relation to socioeconomic levels in Asian populations. Based on the literature from Western countries, certain tendencies have been observed. Navarro et al. [33] reported that, in a Spanish sample, postmenopausal women with poor socioeconomic status had lower BMD values at the lumbar spine and a higher risk of total and vertebral fractures than their counterparts with better socioeconomic status. According to Brennan et al. [34], among Canadian men, the relative risk of osteoporotic fractures for the lowest versus highest income quintile was 1.63 (95% CI 1.42–1.87) and the negative trend was statistically significant (*p* < 0.0001). For women, the risk ratio of fractures for the lowest versus highest income quintile was 1.14 (95% CI 1.01–1.28), with a statistically significant negative trend (*p* = 0.0291). In a Portuguese study, there was a higher risk of osteoporosis in low-income [OR 1.90 (95% CI: 1.07–3.37)] and food-insecure [3.48 (1.43–8.48)] populations. There was a stronger association with food insecurity among women [4.91 (2.40–10.0)] than men [0.46 (0.07–3.01)] [35]. Similarly, in our study, the lower the socioeconomic level, the higher the prevalence of osteoporosis. In a previous South Korean study, income was related to osteoporosis prevalence only in men; however, an inherent limitation of the aforementioned study was that the sample consisted solely of patients who had been diagnosed with osteoporosis [17]. In our study, a nationally representative sample of postmenopausal women aged over 50 was utilized, and we were able to comprehensively identify the role of socioeconomic level, including in those who had not been diagnosed with osteoporosis.

Many studies have reported on the relationship between socioeconomic inequality and chronic diseases. In a South Korean study using National Health Insurance Service data, economic level was related to kidney disease and diabetes [36, 37]. Furthermore, the total risk of cancer in men and women was reported to be 1.65 and 1.43 times higher in the lower income group, respectively [14]. In our study, the prevalence of osteoporosis was 1.6 times higher in the lower income group. Thus, income levels are as relevant in the context of osteoporosis as they are with regard to other diseases.

Population aging is progressing at a rapid rate, resulting in an increased prevalence of diseases characteristic of older adults, such as osteoporosis. The increased social cost of osteoporosis and high mortality from fractures are the primary concerns in this regard, necessitating active prevention [6, 38]. As with other diseases, early detection, education, and awareness can reduce a lot of the social costs associated with osteoporosis and help people prepare for healthy aging. This requires personal efforts as well as national policy. As shown in our study, even after statistical correction for exercise, the prevalence of osteoporosis associated with socioeconomic levels differed. Therefore, it is necessary to close the socioeconomic gap. In our study, the possibility of early detection in the group with low socioeconomic level was low. Based on previous studies and our findings, we recommend the introduction of a national screening program for osteoporosis in women aged over 50.

Despite the aforementioned implications, this study is limited by its use of cross-sectional data, which hindered the examination of the trend of osteoporosis risk according to socioeconomic level. In addition, the relatively small sample size posed a barrier to detailed analyses. In future research, it is necessary to study socioeconomic disparities in the prevalence and diagnosis experience of osteoporosis using a longitudinal design, such as through larger-scale prospective cohort studies.

## Conclusion

The prevalence of osteoporosis increased with age. In addition, low socioeconomic level was associated with a higher risk of developing osteoporosis. Moreover, the lower the socioeconomic level, the lower the awareness of osteoporosis. Therefore, it is necessary to prepare more proactive interventional measures such as national screening for osteoporosis in postmenopausal women with low socioeconomic status.

## Supporting information

S1 Checklist STROBE statement—checklist of items that should be included in reports of observational studies. (DOCX)
